# The Impact of Acute Ammonia Nitrogen Stress on the Gill Tissue Structure and Antioxidant Ability of Gills and Red and White Muscle in Juvenile Yellowfin Tuna (*Thunnus albacares*)

**DOI:** 10.3390/antiox13111357

**Published:** 2024-11-06

**Authors:** Yongyue Sun, Zhengyi Fu, Xuancheng Liu, Zhenhua Ma

**Affiliations:** 1 Key Laboratory of Efficient Utilization and Processing of Marine Fishery Resources of Hainan Province, Sanya Tropical Fisheries Research Institute, Sanya 572018, China; 2 South China Sea Fisheries Research Institute, Chinese Academy of Fishery Sciences, Guangzhou 510300, China; 3 Hainan Engineering Research Center for Deep-Sea Aquaculture and Processing, Sanya Tropical Fisheries Research Institute, Sanya 572018, China; 4 International Joint Research Center for Conservation and Application of Fishery Resources in the South China Sea, Sanya Tropical Fisheries Research Institute, Sanya 572018, China; 5 College of Fisheries and Life Science, Shanghai Ocean University, Shanghai 201306, China; 6 College of Science and Engineering, Flinders University, Adelaide 5001, Australia; 7 College of Life Sciences and Food Engineering, Inner Mongolia Minzu University, Tongliao 028007, China

**Keywords:** NH_3_-N, juvenile yellowfin tuna, gill tissue sections, muscle, antioxidant ability

## Abstract

To explore the impacts of acute ammonia nitrogen (NH_3_-N) stress on gill structure and the antioxidant ability of red and white muscles in juvenile yellowfin tuna (*Thunnus albacares*), this study used natural seawater as a control, establishing two experimental NH_3_-N groups at 5 and 10 mg/L. Gills and red and white muscle were taken at 6, 24, and 36 h for the determination of malondialdehyde (MDA), superoxide dismutase (SOD), catalase (CAT), and glutathione peroxidase (GHS-PX) levels, and to observe gill structure. The results indicated that, with increasing time, the MDA concentration and CAT activity in the gills of the 5 mg/L group showed a trend of first increasing and then decreasing, while SOD activity exhibited a downward trend. In the 10 mg/L group, MDA concentration showed an increasing trend, while SOD, CAT, and GSH-PX activities demonstrated a trend of first increasing and then decreasing. In the 5 mg/L group, the MDA concentration and GSH-PX activity in the red muscle showed an increasing trend. In the 10 mg/L group, MDA concentration and SOD and CAT activities exhibited a downward trend. In the 5 mg/L group, the MDA concentration and SOD activity in the white muscle showed a downward trend, while CAT activity exhibited an increasing trend. In the 10 mg/L group, MDA concentration and CAT activity demonstrated a trend of first increasing and then decreasing, while SOD activity showed a downward trend. Ammonia nitrogen can lead to necrosis and shedding of gill epithelial cells, cell vacuolation, edema, as well as proliferation, hypertrophy, and fusion of secondary lamellae. This study demonstrates that NH_3_-N can alter gill structure and reduce the antioxidant ability of gills and red–white muscle. The findings provide scientific data that can support the aquaculture and recirculating aquaculture systems of juvenile tuna.

## 1. Introduction

Ammonia nitrogen (NH_3_-N) is the final product of the decomposition of fish excrement in aquatic ecosystems, and it is also one of the key factors contributing to the pollution of aquaculture waters [1]. It mainly originates from the metabolic excretion of aquatic organisms, uneaten feed residues, and the discharge of industrial and domestic wastewater, among other sources [2]. In water bodies, ammonia nitrogen exists in two forms: ionic ammonia (NH_4_^+^) and non-ionic ammonia (NH_3_). Ammonia nitrogen can have negative effects on aquatic animals, such as causing an abnormal increase in gill ventilation, impairing body balance, leading to convulsions, disrupting ion balance, and potentially resulting in an overexcited state [3]. Furthermore, ammonia nitrogen can penetrate through the gills and skin into the animal’s body, disrupting cellular functions. The accumulation of ammonia nitrogen damages gill tissues, affects respiration and gas exchange, leads to metabolic disorders, suppresses the immune system, hinders growth and development, and may cause abnormal behavior [4,5,6]. Research shows that it can lead to lamellar deformation and shortening of the gills in clownfish (*Premnas biaculeatus*), as well as hyperplasia and hypertrophy of the mucous cells [7]. Ammonia nitrogen stress may also cause damage to the intestinal tissue structure of barramundi (*Lates calcarifer*) [8]. Ammonia nitrogen can cause oxidative stress in cuttlefish (*Sepia pharaonis*), leading to changes in antioxidant ability [9]. Ammonia nitrogen can promote the enhancement of immune ability in pacific white shrimp (*Litopenaeus vannamei*) [10].

Ammonia nitrogen stress increases the production of reactive oxygen species (ROS) in the body, leading to lipid peroxidation and subsequently elevated levels of the lipid peroxidation product malondialdehyde (MDA), which reduces antioxidant capacity and, in severe cases, may lead to death [6,11,12,13,14]. To counteract oxidative damage caused by ROS, fish activate their antioxidant defense mechanisms. This defense system is primarily composed of key antioxidant enzymes such as superoxide dismutase (SOD), catalase (CAT), and glutathione peroxidase (GSH-PX) [15,16]. SOD plays a crucial role in the bodies of fish, effectively converting harmful superoxide anion radicals into relatively harmless hydrogen peroxide (H_2_O_2_). Subsequently, CAT intervenes to decompose H_2_O_2_ into water and oxygen, thereby further reducing the risk of oxidative damage. GSH-PX is involved in reducing molecular oxygen, water, and lipid hydroperoxides, ensuring that these substances do not damage cells. These three types of enzymes work synergistically to form the core of the fish’s antioxidant defense system, protecting fish from oxidative stress and maintaining their health [12,15,17]. Studies have shown that under ammonia nitrogen stress, the concentration of MDA in the body of cuttlefish increases, while the activities of SOD and CAT decrease [9]. Under acute ammonia nitrogen stress, the SOD activity in the liver of juvenile yellowfin tuna (*Thunnus albacares*) showed a decreasing trend [18]. It is evident that long-term exposure to ammonia nitrogen environments can impact the antioxidant systems of aquatic animals, thereby reducing their resistance to diseases. This, in turn, can have implications for the health and stability of the entire aquatic ecosystem.

Yellowfin tuna is the most economically valuable species within the genus Thunnus, known for its high protein and low fat concentration, making it a healthy food choice that is highly favored by consumers [19]. As a fast, continuously swimming large midwater and epipelagic bony fish, the tuna swims in tropical and subtropical warm waters, requiring a large amount of oxygen due to its swimming habits [20,21,22]. Their locomotor ability and energy metabolism are closely related to skeletal muscles. Skeletal muscles are key tissues for maintaining overall metabolism and movement in the body, and they can be primarily divided into two main types based on their functions: white muscle and red muscle [23,24]. As migratory fish, yellowfin tuna require more fat, glycogen, and myoglobin for long-distance travel, which results in a higher proportion of red muscle compared to white muscle, reaching up to 48%. The characteristics of this muscle tissue enable yellowfin tuna to swim rapidly for extended periods, which is a crucial physiological feature for adapting to marine environments and completing their life cycle [25]. Therefore, there are numerous differences between the two types of muscle found in tuna [25]. Reports indicate that international laboratories in Panama and Indonesia, among others, have conducted research on the artificial cultivation of yellowfin tuna [26,27]; currently, yellowfin tuna are affected by overfishing [28]. To date, there has been limited public disclosure of data on the aquaculture of yellowfin tuna, which has restricted the development of its artificial cultivation.

Studying the impact of ammonia nitrogen on the gills, red muscle, and white muscle is crucial for the aquaculture industry. It not only aids in optimizing farming management, enhancing fish survival rates and growth performance, but also reduces disease incidence and enhances the fish’s stress resistance. The outcomes of these studies can improve the quality of farmed products, ensuring food safety, and have a profound impact on maintaining ecological balance and promoting sustainable development.

## 2. Materials and Methods

### 2.1. Material Source

This investigation employed a total of 180 juvenile yellowfin tuna sourced from the wild, with an average weight of 266.28 ± 56.02 g and an average body length of 23.61 ± 2.03 cm. These juveniles were supplied by the Sanya Tropical Fisheries Research Institute which is located in Lingshui County, Hainan Province, China. After the juvenile fish were transferred from the net cages to indoor recirculating aquaculture tanks equipped with a seawater filtration system, they underwent a 7-day acclimation period, they underwent a one-week acclimation period during which they were fed with fresh frozen mixed fish. Throughout the culture period, water quality was maintained within an ideal range, with water temperature kept at 29 ± 1 °C, ammonia nitrogen concentration below 0.01 milligrams per liter, dissolved oxygen concentration maintained at 7 ± 0.5 milligrams per liter, pH value at 8.1, salinity at 32‰, and nitrite concentration below 0.01 mg/L.

### 2.2. Experimental Design and Method

Based on previous experimental results, a stress time of 36 h was selected, and three appropriate concentrations were chosen for the experiment [29,30,31]. The experiment used natural seawater as the control group, with an ammonia nitrogen concentration of 0 mg/L. The ammonia nitrogen experimental groups were set at 5 and 10 mg/L, resulting in three experimental groups, each with three replicates. Analytical grade ammonium chloride (NH_4_Cl) (purity of 99.5%, Xilong Chemical Co., Ltd., Foshan, China) was prepared as a stock solution at 10 g/L to serve as a source of ammonia nitrogen and was added to the tank, with the filtration system turned off. At the start of the experiment, juvenile yellowfin tuna were randomly transferred to nine recirculating aquaculture tanks, each with a volume of 5000 L. Each tank housed 20 juvenile yellowfin tuna. Water quality indicators were controlled as during the acclimation period, and feeding was not conducted during the experiment. The mass concentration of ammonia nitrogen was measured every two hours using a portable water quality tester (provided by Wuxi Aokedian Biotechnology Co., Ltd., Wuxi, China). The mass concentration of ammonia nitrogen was supplemented in a timely manner. At 6, 24, and 36 h, three yellowfin tuna were randomly selected from each treatment group for measurement.

### 2.3. Sample Collection

After anesthetizing the juvenile yellowfin tuna with eugenol (Shangchi Dental Material Co., Ltd., Changshu, China), the body weight was measured and the body length recorded. Gill filaments were placed in a centrifuge tube containing 4% paraformaldehyde (500 mL, Langjieke Technology Co., Ltd., Hefei, China), ensuring that the filaments were submerged in paraformaldehyde and stored at room temperature for subsequent sectioning. The gills, red muscle, and white muscle were placed in cryopreservation tubes and immediately immersed in liquid nitrogen for rapid freezing. The next day, they were transferred to a −80 °C freezer for storage, to be used in later experimental analyses.

### 2.4. Assay of Enzyme Activity

Tissue weighing 0.1–0.2 g was placed in a 2 mL centrifuge tube, along with 2–3 steel beads. A 0.9% saline solution was added at a mass (g) to volume (mL) ratio of 1:9. The mixture was homogenized using a homogenizer (Hangzhou Aosheng Instrument Co., Ltd., Hangzhou, China), and then centrifuged at 3500 r for 10 min using a centrifuge (Biofuge Company, Hanau, Germany).

The enzyme activity measurements in this experiment were obtained from commercial kits produced by the Nanjing Jiancheng Bioengineering Institute (Nanjing, China). MDA levels were quantified using an MDA assay kit (catalog number: A003-1). As a byproduct of lipid peroxidation, MDA reacts with thiobarbituric acid (TBA) to form a red compound, with absorbance recorded at 532 nm for analysis. SOD activity was evaluated using the water-soluble tetrazolium salt (WST-1) method with a SOD assay kit (catalog number: A001-3). CAT activity was measured using a CAT assay kit (catalog number: A007-1) via the ammonium molybdate method. GSH-PX activity was assessed with a GSH-PX assay kit (catalog number: A005-1), where GSH-PX activity is represented by the catalytic rate of GSH reaction.

### 2.5. Gill Histology Observation

The gill filaments of juvenile yellowfin tuna were fixed in 4% paraformaldehyde solution, followed by dehydration, clearing, and embedding in paraffin. They were then sectioned into 4-micron-thick cross-sections using a Leica RM 2016 rotary microtome (Leica Instruments Shanghai Co., Ltd., Shanghai, China). These sections were stained with hematoxylin and eosin (HE) for microscopic observation of their tissue structure. The stained gill tissue sections were mounted on slides using neutral resin (Beijing Solarbio Science & Technology Co., Ltd., Beijing, China) for preservation. The sections were observed, photographed, and saved using an inverted biological microscope (DMI8, Leica, Wetzlar, Germany), and were analyzed according to the literature [32,33]. The average prevalence of histopathological parameters in gills is classified into three levels: mild (+, <25% of sections), moderate (++, 25–50% of sections), and severe (+++, >50% of sections).

### 2.6. Statistical Analysis

In this experiment, the experimental data are presented as mean ± standard deviation (mean ± SD). One-way ANOVA was conducted on different ammonia nitrogen concentrations at the same time using SPSS 25.0 (2025 edition, IBM, Armonk, NY, USA), and two-way ANOVA was performed for time and ammonia nitrogen concentration. Before analysis, the data were tested for normal distribution and homogeneity of variance. If significant differences were found (*p* < 0.05), one-way ANOVA using the Duncan method was applied to compare different ammonia nitrogen concentrations at the same time. When there were extremely significant differences (*p* < 0.01) or significant differences (*p* < 0.05), letters a, b, and c were used to indicate differences between the control group, 5 mg/L, and 10 mg/L at the same time. Origin 2022 (2022 edition) (OriginLab, Northampton, MA, USA) was used for plotting the experimental data.

## 3. Result

### 3.1. Microscopic Observation of Juvenile Yellowfin Tuna Gill Tissue

**Figure 1:**
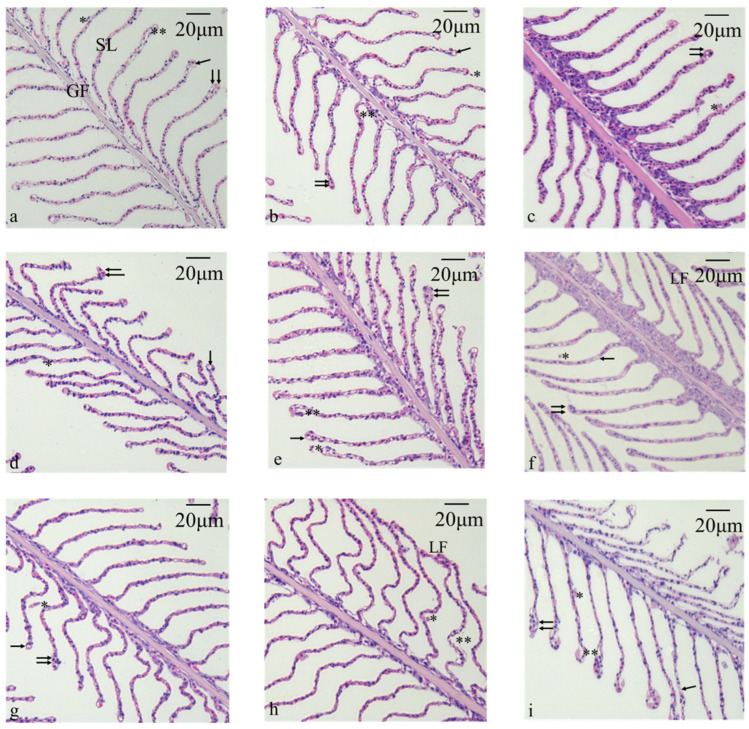
Microscopic observation of juvenile yellowfin tuna gill tissue structure under acute ammonia nitrogen stress (*n* = 9). (**a**) 0 mg/L stress of 6 h; (**b**) 0 mg/L stress of 24 h; (**c**) 0 mg/L stress of 36 h; (**d**) 5 mg/L stress of 6 h; (**e**) 5 mg/L stress of 24 h; (**f**) 5 mg/L stress of 36 h; (**g**) 10 mg/L stress of 6 h; (**h**) 10 mg/L stress of 24 h; (**i**) 10 mg/L stress of 36 h. GF—gill filament; SL—secondary lamellae; LF—lamellae fusion; (*) epithelial necrosis and desquamation; (**) epithelial cell edema; (↑) cellular vacuolation; (↑↑) proliferation and hypertrophy of secondary lamellae.

The gills of juvenile yellowfin tuna primarily consist of gill filaments and secondary lamellae. The secondary lamellae are thin, plate-like structures attached to both sides of the interlamellar spaces, typically arranged symmetrically and in abundance (Figure 1). In the control group, mild edema of epithelial cells was observed at 6 h, accompanied by moderate levels of epithelial cell necrosis, shedding, and vacuolation. Additionally, proliferation and hypertrophy of secondary lamellae were also noted (Figure 1a). In the control group, at 24 h, mild epithelial cell necrosis and shedding were observed, along with moderate epithelial cell edema, vacuolation, and proliferation and hypertrophy of secondary lamellae (Figure 1b). In the control group, at 36 h, moderate epithelial cell necrosis and shedding, as well as proliferation and hypertrophy of secondary lamellae, were observed (Figure 1c). In the 5 mg/L group, at 6 h, mild epithelial cell vacuolation was observed, along with moderate epithelial cell necrosis, shedding, and proliferation and hypertrophy of secondary lamellae (Figure 1d). In the 5 mg/L group, at 24 h, mild epithelial cell edema was observed, along with moderate epithelial cell necrosis, shedding, vacuolation, and proliferation and hypertrophy of secondary lamellae (Figure 1e). In the 5 mg/L group, at 36 h, mild necrosis, desquamation, and vacuolation of epithelial cells was observed, as well as proliferation and hypertrophy of secondary lamellae, with severe lamellae fusion (Figure 1f). In the 10 mg/L group, at 6 h, mild epithelial cell necrosis, shedding, and vacuolation were observed, along with moderate proliferation and hypertrophy of secondary lamellae (Figure 1g). In the 10 mg/L group, at 24 h, mild epithelial cell edema and lamellar fusion were observed, along with moderate epithelial cell necrosis and shedding (Figure 1h). In the 10 mg/L group, at 36 h, mild epithelial cell vacuolation was observed, while epithelial cell edema, necrosis, shedding, and proliferation and hypertrophy of secondary lamellae were severe (Figure 1i). The summary of the observed histopathological changes in the gills is detailed in Table 1.

### 3.2. Effect of Ammonia Nitrogen on Gill Antioxidant Ability

The homogeneity of variance of MDA concentration data in gills is shown in Table S1 (*p* > 0.05). The interaction between ammonia nitrogen concentration and time has a significant impact on the MDA concentration of the gills (*p* < 0.05, Table S2). As time went on, the 5 mg/L treatment group showed an initial increase and then a decrease, reaching a peak at 24 h with a concentration of 13.19 ± 0.24 nmol/mgprot. At 6 and 24 h, the MDA concentration in the 5 mg/L group was significantly higher than that in the control group (*p* < 0.01, Table 2), while at 36 h, it was significantly lower than in the control group (*p* < 0.01). In the 10 mg/L treatment group, the MDA concentration showed an increasing trend over time, reaching its maximum at 36 h (13.57 ± 0.08 nmol/mg protein), and was significantly higher than that in the control group at 36 h (*p* < 0.01).

The homogeneity of variance of SOD activity data in gills is shown in Table S3 (*p* > 0.05). The interaction between ammonia nitrogen concentration and time has a significant impact on the SOD activity of the gills (*p* < 0.05, Table S4). Over time, the gills of juvenile yellowfin tuna exposed to 5 mg/L of ammonia nitrogen displayed a fluctuating trend in SOD activity, initially decreasing to a nadir at 24 h with a value of 5.42 ± 0.90 U/mgprot, followed by an increase. In contrast, the gills of the 10 mg/L treatment group showed an initial surge in SOD activity, peaking at 24 h with a value of 8.66 ± 1.63 U/mgprot, before subsequently decreasing. The SOD activity in the 5 mg/L group was significantly higher than that in the control group at 6 h (*p* < 0.01, Table 2) and significantly higher at 36 h (*p* < 0.05). From 6 to 36 h, there was no significant difference in SOD activity between the 10 mg/L group and the control group (*p* > 0.05).

The homogeneity of variance of CAT activity data in gills is shown in Table S5 (*p* > 0.05). The interaction between ammonia nitrogen concentration and time has a significant impact on the CAT activity of the gills (*p* < 0.05, Table S6). The CAT activity in the gills of the ammonia nitrogen-exposed group exhibited a temporal pattern of initial elevation followed by a subsequent decline, peaking at 24 h with values of 19.68 ± 1.06 U/mgprot and 31.32 ± 0.86 U/mgprot, respectively. At 24 h, the CAT activity in the ammonia nitrogen experimental group was significantly higher than that in the control group (*p* < 0.01, Table 2).

The homogeneity of variance of GSH-PX activity data in gills is shown in Table S7 (*p* > 0.05). The interaction between ammonia nitrogen concentration and time has a significant impact on the GSH-PX activity of the gills (*p* < 0.05, Table S8). As time progresses, the activity of GSH-PX in the 5 mg/L treatment group remained relatively stable. In contrast, in the 10 mg/L treatment group, it initially showed an increase followed by a decrease, reaching a peak at 24 h with an activity level of 89.88 ± 2.24 units. The GSH-PX activity in the 5 mg/L treatment group was significantly lower than that in the control group at 24 h (*p* < 0.01, Table 2). The GSH-PX activity in the 10 mg/L treatment group was significantly lower than that in the control group throughout the experiment (*p* < 0.01).

### 3.3. Effect of Ammonia Nitrogen on Red Muscle Antioxidant Ability

The homogeneity of variance of MDA concentration data in red muscle is shown in Table S9 (*p* > 0.05). The interaction between ammonia nitrogen concentration and time has a significant impact on the MDA concentration of the red muscle (*p* < 0.05, Table S10). As time passed, the red muscle of the 5 mg/L treatment group exhibited an increase in MDA concentration, reaching a peak value of 1.60 ± 0.09 nmol/mgprot at 36 h. In contrast, the red muscle of the 10 mg/L treatment group showed a decreasing trend in MDA concentration, reaching its lowest point at 36 h with a value of 0.70 ± 0.01 nmol/mgprot. The MDA concentration in the red muscle of the 5 mg/L treatment group was significantly lower than that in the control group at 6 and 24 h (*p* < 0.01, Table 3), but significantly higher at 36 h (*p* < 0.01). In the 10 mg/L treatment group, the MDA concentration in the red muscle was significantly higher than that in the control group at 6 h (*p* < 0.05), significantly lower at 24 h (*p* < 0.01), and significantly lower at 36 h (*p* < 0.05).

The homogeneity of variance of SOD activity data in red muscle is shown in Table S11 (*p* > 0.05). The interaction between ammonia nitrogen concentration and time has a significant impact on the SOD activity of the red muscle (*p* < 0.05, Table S12). Over time, the SOD activity in the ammonia nitrogen treatment group showed a declining trend, reaching its lowest point at 36 h with values of 0.27 ± 0.04 U/mgprot and 0.46 ± 0.04 U/mgprot, respectively. At 24 h, the SOD activity in the ammonia nitrogen treatment group was significantly higher than that in the control group (*p* < 0.01, Table 3).

The homogeneity of variance of CAT activity data in red muscle is shown in Table S13 (*p* > 0.05). The interaction between ammonia nitrogen concentration and time has a significant impact on the CAT activity of the red muscle (*p* < 0.05, Table S14). As time elapsed, the CAT activity in the 5 mg/L treatment group exhibited a trend of initially decreasing and then increasing, reaching its minimum at 24 h with a value of 0.24 ± 0.07 U/mgprot. In contrast, the CAT activity in the 10 mg/L treatment group showed a continuous decline, reaching its lowest point at 36 h with a value of 0.71 ± 0.09 U/mgprot. The CAT activity in the red muscle of the 5 mg/L treatment group was significantly lower than that in the control group at 6 and 24 h (*p* < 0.01, Table 3). In the 10 mg/L treatment group, the CAT activity in the red muscle was significantly higher than that in the control group at 6 h (*p* < 0.01). At 36 h, there was no significant difference in CAT activity between the ammonia nitrogen treatment group and the control group (*p* > 0.05).

The homogeneity of variance of CAT activity data in red muscle is shown in Table S15 (*p* > 0.05). The interaction between ammonia nitrogen concentration and time has a significant impact on the GSH-PX activity of the red muscle (*p* < 0.05, Table S16). As time marches on, the activity of GSH-PX in the red muscle of the 5 mg/L treatment group progressively increased, attaining a peak at 36 h with an activity level of 13.21 ± 0.64 units. In contrast, the GSH-PX activity in the red muscle of the 10 mg/L treatment group displayed a decreasing tendency, reaching its lowest point at 36 h with an activity level of 7.11 ± 0.31 units. The GSH-PX activity in the red muscle of the 5 mg/L treatment group was significantly higher than that in the control group at 36 h (*p* < 0.01, Table 3). The GSH-PX activity in the red muscle of the 10 mg/L treatment group was significantly higher than that in the control group at both 6 and 36 h (*p* < 0.01).

### 3.4. Effect of Ammonia Nitrogen on White Muscle Antioxidant Ability

The homogeneity of variance of MDA concentration data in white muscle is shown in Table S17 (*p* > 0.05). The interaction between ammonia nitrogen concentration and time has a significant impact on the MDA concentration of the white muscle (*p* < 0.05, Table S18). As time advances, the MDA concentration in the white muscle of the 5 mg/L treatment group exhibited a decreasing trend, reaching a minimum value of 0.27 ± 0.01 nmol/mgprot at 36 h. In contrast, the MDA concentration in the white muscle of the 10 mg/L treatment group demonstrated a pattern of initially decreasing and then increasing, peaking at 24 h with a value of 0.48 ± 0.014 nmol/mgprot. The MDA concentration in the white muscle of the 5 mg/L treatment group was significantly lower than that in the control group at 24 and 36 h (*p* < 0.01, Table 4). In the 10 mg/L treatment group, the MDA concentration was significantly higher than that in the control group at 24 h (*p* < 0.01).

The homogeneity of variance of SOD activity data in white muscle is shown in Table S19 (*p* > 0.05). The interaction between ammonia nitrogen concentration and time has a significant impact on the SOD activity of the white muscle (*p* < 0.05, Table S20). With the increase in time, the SOD activity in both the 5 mg/L and 10 mg/L treatment groups exhibited a decreasing trend, reaching a minimum at 36 h with values of 0.17 ± 0.02 U/mgprot and 0.19 ± 0.02 U/mgprot, respectively. The SOD activity in the white muscle of the 5 mg/L group was significantly lower than that in the control group at 36 h (*p* < 0.05, Table 4), while the SOD activity in the 10 mg/L group showed no significant difference compared to the control group (*p* > 0.05).

The homogeneity of variance of CAT activity data in white muscle is shown in Table S21 (*p* > 0.05). The interaction between ammonia nitrogen concentration and time has a significant impact on the CAT activity of the white muscle (*p* < 0.05, Table S22). As time progresses, the CAT activity in the white muscle of the 5 mg/L treatment group exhibited an increase and attained a maximum activity value of 0.61 ± 0.12 U/mgprot at 36 h. In contrast, in the 10 mg/L treatment group, the CAT activity in the white muscle initially ascended and then declined, reaching a peak at 24 h with an activity value of 0.35 ± 0.10 U/mgprot. The CAT activity in the white muscle of the 5 mg/L treatment group was significantly lower than that in the control group at 6 h (*p* < 0.01, Table 4), significantly lower at 24 h (*p* < 0.05), and significantly higher at 36 h (*p* < 0.01). In the 10 mg/L treatment group, the CAT activity in the white muscle was significantly lower than that in the control group at 6 h (*p* < 0.01) and 24 h (*p* < 0.05), while there was no significant difference at 36 h (*p* > 0.05).

The homogeneity of variance of GSH-PX activity data in white muscle is shown in Table S23 (*p* > 0.05). The interaction between ammonia nitrogen concentration and time has a significant impact on the GSH-PX activity of the white muscle (*p* < 0.05, Table S24). The white muscle of the 5 mg/L treatment group demonstrated a decreasing trend in GSH-PX activity over time, reaching a minimum of 10.23 ± 2.23 activity units at 24 h. This decline peaked at 6 h. In contrast, at 24 h, the GSH-PX activity in the white muscle of the 10 mg/L treatment group began to rise, peaking at 36 h with a measurement of 28.50 ± 2.23 activity units. At 6 and 24 h, there were no significant differences among the three groups (*p* > 0.05, Table 4). At 36 h, the GSH-PX activity in the white muscle of the 5 mg/L treatment group was significantly lower than that in the control group (*p* < 0.05), while the GSH-PX activity in the white muscle of the 10 mg/L treatment group was significantly higher than that in the control group (*p* < 0.01).

## 4. Discussion

### 4.1. Effects of Acute Ammonia Nitrogen Stress on the Gill Tissue Structure of Juvenile Yellowfin Tuna

As the key site for gas exchange in fish, the gill tissue structure is highly sensitive to even minor changes in the aquatic environment. Any pathological changes in the gill tissue can have profound effects on the vital physiological activities of aquatic animals, including feeding, respiration, excretion, and the regulation of acid–base and ionic balance [34,35,36]. After ammonia nitrogen stress, the gill filaments of golden pompano (*Trachinotus ovatus*) gradually become shorter, curled, and fused, with tissue damage progressively worsening over time [37]. In loach (*Misgurnus anguillicaudatus*) and grass carp (*Ctenopharyngodon idella*), both long-term and short-term ammonia nitrogen stress can cause edema and even necrosis in the epithelial cells and chloride cells of the gill tissue [36,38]. In this study, the 5 mg/L group showed no significant damage or inflammatory response in gill cells during the 6 h and 24 h stress phases. However, as the stress duration was extended to 36 h, the effects of ammonia nitrogen on gill cells gradually became apparent, leading to intercellular fusion. This may be due to ammonia nitrogen inhibiting the respiration of juvenile yellowfin tuna. In the 10 mg/L treatment group, lamellar fusion was observed after 24 h of exposure. This may be due to ammonia nitrogen limiting the respiration of juvenile yellowfin tuna. The fusion of gill filaments reduces the surface area of the gills, thereby decreasing the efficiency of gas exchange. As exposure time extended to 36 h, severe epithelial cell edema, necrosis, shedding, and proliferation and hypertrophy of secondary lamellae were observed, along with the dissolution of gill cell nuclei, a reduction in cell numbers, and the blurring of the edges of the gill cells [39]. The high concentration of ammonia nitrogen and prolonged stress may have caused damage to gill cells not only morphologically but also in terms of their cellular structure and function. The dissolution of cell nuclei and the reduction in cell numbers directly affect the survival and regenerative capacity of the cells, while the blurring of cell edges may be a result of impaired intercellular junctions, further weakening the overall function of the gills. Ammonia nitrogen can cause damage and sloughing of the gill epithelial cells in juvenile yellowfin tuna, and can also lead to fusion of the gill filaments, thereby reducing gas exchange efficiency and affecting the respiratory function of the fish.

### 4.2. Effects of Acute Ammonia Nitrogen Stress on Antioxidant Ability of Gill of Juvenile Yellowfin Tuna

The gills are not only important organs for aquatic animals to ingest food and perform respiratory functions, but they also play a significant role in osmoregulation within the animal’s body [40,41]. Studies have shown that under acute ammonia nitrogen stress, the SOD activity in the gills of barramundi tends to decrease [8]. In contrast, under other stress conditions, the SOD activity in the gills of juvenile yellowfin tuna tends to increase under salinity stress, with MDA levels peaking towards the later stages of stress [26]. In this experiment, at 36 h, the concentration of MDA in the gills in the 5 mg/L group was lower than that in the control group, while the SOD activity was higher than that of the control group. This may be due to the fact that low concentrations of ammonia nitrogen have a stimulatory effect on SOD activity. The increase in SOD activity inhibits the production of ROS in the gills of juvenile yellowfin tuna, thereby reducing lipid peroxidation in the gills and consequently lowering the production of MDA. At 36 h, in the 10 mg/L treatment group, the MDA concentration in the gills of juvenile yellowfin tuna was higher than that of the control group, while the activities of SOD and CAT showed no significant difference compared to the control group. The activity of GSH-PX was lower than that of the control group. This might suggest that higher concentrations of ammonia nitrogen aggravate the damage of lipid peroxidation in the gills, leading to an increase in MDA concentration. At the same time, high concentrations of ammonia nitrogen may have a negative impact on the antioxidant system in the gills, with little effect on the activities of SOD and CAT, but inhibiting the activity of GSH-PX. These results disclose that ammonia nitrogen diminishes the influence of the antioxidant defense system in the gills of juvenile yellowfin tuna.

### 4.3. Effects of Acute Ammonia Nitrogen Stress on Antioxidant Ability of Red Muscle of Juvenile Yellowfin Tuna

The muscle of the yellowfin tuna can be divided into white muscle and red muscle [42]. The red muscle is less abundant in the fish’s trunk, located in a superficial longitudinal band under the skin. When stimulated, the red muscle has a slow response speed and contraction speed, but a long duration, and is therefore known as slow-twitch muscle [43,44]. Red muscle primarily relies on aerobic metabolism for sustained muscle contraction and has a higher aerobic metabolic ability than white muscle [45,46,47]. The cuttlefish exhibits a declining trend in the activities of SOD and CAT when exposed to ammonia nitrogen stress [9]. The phenomenon of lipid peroxidation can be exacerbated by ammonia nitrogen in both the cuttlefish and juvenile sturgeon (*Acipenser schrenckii*), which has been shown by an increase in MDA concentration in both species [9,48]. Other environmental stress studies have shown that in juvenile yellowfin tuna, the red muscle’s MDA concentration, SOD, and CAT activity do not change significantly under low pH stress, while GSH-PX activity tends to increase with decreasing pH [49]. The experimental results indicate that in the 5 mg/L group, after 6 h of stress, the SOD activity in red muscle increased, reducing MDA production. GSH-PX has a higher affinity for H_2_O_2_ than CAT, thus GSH-PX eliminated the produced H_2_O_2_ while CAT activity was suppressed [50,51]. After 36 h of stress, excess ammonia nitrogen accumulated in the juvenile fish, leading to the production of a large amount of MDA. The remaining SOD decomposed this MDA, generating H_2_O_2_, which induced an increase in the activity of CAT and GSH-PX to decompose H_2_O_2_. In the 10 mg/L group, the MDA concentration and the activities of SOD and CAT in the red muscle of juvenile fish showed a downward trend, indicating that ammonia nitrogen caused more severe damage to the red muscle of juvenile yellowfin tuna, significantly impairing their antioxidant system. It is evident that high concentrations of ammonia nitrogen cause more severe oxidative damage to yellowfin tuna compared to lower concentrations. Although CAT and GSH-PX have similar functions, their trends of change in this experiment were different. GSH-PX may independently eliminate peroxides [52]. Studying the damage of ammonia nitrogen to the antioxidant ability of red muscle in juvenile yellowfin tuna is crucial for understanding the environmental adaptability of fish, preventing diseases, protecting juvenile fish, and improving the quality of fish products.

### 4.4. Effects of Acute Ammonia Nitrogen Stress on Antioxidant Ability of White Muscle of Juvenile Yellowfin Tuna

White muscle is located in the deep layer of the muscle segment. When stimulated, white muscle reacts quickly and contracts rapidly, but the duration is short, earning it the name “fast muscle”. White muscle primarily relies on anaerobic metabolism supported by glycolysis for energy to perform explosive movements [43,44,47]. Exposure to 5mg/L of ammonia nitrogen significantly increased the activity of MDA, SOD, CAT, and GSH-PX in the body kidney of yellowfin tuna juveniles compared to the control group after 36 h [53]. In other environmental stress studies, as the concentration of p-Xylene increased, the MDA concentration and SOD activity in the white muscle of the *Boleophthalmus pectinirostris* increased, while the GSH-PX activity decreased [54]. In this experiment, as time increased, the MDA concentration in the white muscle in the 5 mg/L group showed a decreasing trend, while the SOD activity exhibited a decrease–increase trend, and the CAT activity showed an increasing trend. The GSH-PX activity did not change significantly. This may be due to the fact that at the beginning of the stress, the white muscle underwent lipid peroxidation under the stress of 5 mg/L ammonia nitrogen. As the stress time extended, the 5 mg/L ammonia nitrogen inhibited the aerobic respiration of the white muscle, leading to a decrease in reactive oxygen species and thus a reduction in MDA concentration. The decrease in SOD activity corresponds with the decrease in MDA concentration at 24 h of stress. As the stress time continued, the body’s antioxidant ability improved, resulting in an increase in SOD activity and CAT. GSH-PX, due to its lagging effect, showed little change. In the 10 mg/L treatment group, the SOD activity remained relatively stable, while the CAT activity showed a “increase-decrease” trend. The GSH-PX activity peaked at 36 h of stress. At 24 h of stress, the 10 mg/L ammonia nitrogen concentration enhanced the body’s antioxidant ability. Subsequently, the antioxidant system was damaged, causing a decrease in CAT activity. At this point, GSH-PX began to function, with its activity increasing. Consequently, the 10 mg/L ammonia nitrogen concentration exceeds the tolerance range of yellowfin tuna white muscle to ammonia nitrogen. During the early and middle stages of stress, it severely disrupts the body’s antioxidant system, leading to minimal changes in SOD, CAT, and GSH-PX activities. Given the lag in GSH-PX response, its activity increases at the 36 h stress mark.

Red muscle, which contains a high number of mitochondria for aerobic respiration, is more susceptible to the production of MDA under ammonia nitrogen stress compared to white muscle [45,46]. Red muscle has better oxidative enzyme levels than white muscle, hence its antioxidant enzyme activity is higher than that of white muscle [55]. It is evident that ammonia nitrogen causes different levels of damage to red and white muscles.

## 5. Conclusions

This study set up three ammonia nitrogen concentrations of 0, 5, and 10 mg/L, and randomly selected juvenile yellowfin tuna at 6, 24, and 36 h of stress for the determination of gill tissue structure and antioxidant ability of the gills, red muscle, and white muscle, aiming to measure the impact of ammonia nitrogen on the gill tissue structure and antioxidant ability of the gills and muscles in juvenile yellowfin tuna. Ammonia nitrogen causes changes in the structure of gill tissue, including necrosis and shedding of epithelial cells, cellular vacuolation, and edema, as well as proliferation, hypertrophy, and fusion of secondary lamellae. Damage to the gill tissue may reduce the oxygen consumption of the fish. The oxidative damage to the gills showed an upward trend with 10 mg/L of ammonia nitrogen, and the antioxidant ability of red muscle was stronger than that of white muscle. Therefore, in actual production, to mitigate the oxidative stress on the gills, red muscle, and white muscle of juvenile yellowfin tuna and maintain the health of these tissues, it is recommended that when the ammonia nitrogen concentration ranges from 5 mg/L to 10 mg/L, the stress duration should not exceed 24 h. Meanwhile, when the ammonia nitrogen concentration is lower than 5 mg/L, the stress duration should not exceed 36 h. This is of significant importance for the sustainable development of the yellowfin tuna industry and practical production.

## Figures and Tables

**Table 1:** The impact of ammonia nitrogen on the gill tissue structure of juvenile yellowfin tuna.

Parameters	0 mg/L			5 mg/L			10 mg/L		
	6 h	24 h	36 h	6 h	24 h	36 h	6 h	24 h	36 h
Epithelial cell edema	+	++	-	-	+	-	-	+	+++
Epithelial necrosis and desquamation	++	+	++	++	++	+	+	++	+++
Cellular vacuolation	++	++	-	+	++	+	+	-	+
Lamellae fusion	-	-	-	-	-	+++	-	+	-
Proliferation and hypertrophy of secondary lamellae	++	++	++	++	++	+	++	-	+++

(-) None, (+) mild, (++) moderate, (+++) severe.

**Table 2:** Effects of acute ammonia nitrogen stress on the antioxidant ability of gills of juvenile yellowfin tuna (n = 9).

Enzyme Activity	Time (h)	Ammonia Nitrogen Concentration (mg/L)		
		0	5	10
MDA	6	3.33 ± 0.21 ^b^	6.94 ± 0.17 ^a^	3.32 ± 0.01 ^b^
	24	8.34 ± 0.12 ^b^	13.19 ± 0.24 ^a^	7.91 ±0.01 ^c^
	36	11.76 ± 0.12 ^b^	8.14 ± 0.11 ^c^	13.57 ± 0.08 ^a^
SOD	6	7.60 ± 2.85 ^b^	14.37 ± 1.21 ^a^	7.15 ± 1.06 ^b^
	24	8.85 ± 2.28 ^a^	5.42 ± 0.90 ^a^	8.68 ± 1.63 ^a^
	36	8.09 ± 1.52 ^b^	11.07 ± 1.44 ^b^	6.22 ± 0.83 ^b^
CAT	6	5.08 ± 0.93 ^b^	8.35 ± 1.44 ^a^	4.24 ± 0.67 ^b^
	24	7.06 ± 1.75 ^c^	19.68 ± 1.06 ^b^	31.32 ± 0.86 ^a^
	36	10.57 ± 0.79 ^ab^	7.75 ± 3.09 ^b^	14.42 ± 1.44 ^a^
GSH-PX	6	99.49 ± 13.92 ^a^	86.47 ± 9.79 ^a^	64.23 ± 2.51 ^b^
	24	114.14 ± 7.41 ^a^	90.20 ± 7.09 ^b^	89.88 ± 2.24 ^b^
	36	88.12 ± 8.92 ^a^	89.35 ± 4.38 ^a^	23.03 ± 0.89 ^b^

Notes: malondialdehyde (MDA); superoxide dismutase (SOD); catalase (CAT); glutathione peroxidase (GSH-PX). The letters annotated by peers indicate significant differences between different ammonia concentrations at the same time (*p* < 0.05).

**Table 3:** Effects of acute ammonia nitrogen stress on the antioxidant ability of the red muscle of juvenile yellowfin tuna (n = 9).

Enzyme Activity	Time (h)	Ammonia Nitrogen Concentration (mg/L)		
		0	5	10
MDA	6	0.75 ± 0.01 ^b^	0.45 ± 0.01 ^c^	1.02 ± 0.07 ^a^
	24	0.98 ±0.02 ^a^	0.55 ± 0.01 ^c^	0.745 ± 0.01 ^b^
	36	0.87 ± 0.02 ^b^	1.60 ± 0.09 ^a^	0.70 ± 0.01 ^c^
SOD	6	0.54 ±0.11 ^b^	1.13 ± 0.26 ^a^	0.92 ±0.08 ^a^
	24	0.24 ± 0.04 ^c^	0.41 ± 0.02 ^b^	0.50 ± 0.04 ^a^
	36	0.30 ± 0.14 ^a^	0.27 ± 0.04 ^b^	0.46 ±0.04 ^a^
CAT	6	1.16 ± 0.14 ^b^	0.67 ± 0.05 ^c^	1.58 ± 0.09 ^a^
	24	1.15 ± 0.19 ^a^	0.24 ± 0.07 ^b^	0.96 ± 0.03 ^a^
	36	0.64 ± 0.06 ^a^	0.88 ± 0.22 ^a^	0.71 ± 0.093 ^a^
GSH-PX	6	4.28 ± 1.15 ^b^	4.40± 0.06 ^b^	7.60 ± 0.41 ^a^
	24	6.51 ± 0.68 ^ab^	5.86 ± 0.28 ^b^	7.09 ± 0.28 ^a^
	36	2.90 ± 0.78 ^c^	13.21 ± 0.64 ^a^	7.11 ± 0.31 ^b^

Notes: malondialdehyde (MDA); superoxide dismutase (SOD); catalase (CAT); glutathione peroxidase (GSH-PX). The letters annotated by peers indicate significant differences between different ammonia concentrations at the same time (*p* < 0.05).

**Table 4:** Effects of acute ammonia nitrogen stress on the antioxidant ability of the white muscle of juvenile yellowfin tuna (n = 9).

Enzyme Activity	Time (h)	Ammonia Nitrogen Concentration (mg/L)		
		0	5	10
MDA	6	0.48 ± 0.03 ^a^	0.39 ± 0.03 ^ab^	0.38 ± 0.06 ^b^
	24	0.41 ± 0.02 ^b^	0.31 ± 0.01 ^c^	0.48 ± 0.01 ^a^
	36	0.41 ± 0.03 ^a^	0.27 ± 0.01 ^c^	0.31 ± 0.01 ^b^
SOD	6	0.23 ± 0.01 ^a^	0.25 ± 0.03 ^a^	0.27 ± 0.04 ^a^
	24	0.28 ± 0.06 ^a^	0.22 ± 0.03 ^a^	0.20 ± 0.03 ^a^
	36	0.23 ± 0.04 ^a^	0.17 ± 0.02 ^a^	0.19 ± 0.02 ^a^
CAT	6	0.66 ± 0.23 ^a^	0.11 ±0.06 ^b^	0.06 ± 0.04 ^b^
	24	0.97 ± 0.39 ^a^	0.47 ± 0.06 ^b^	0.35 ± 0.10 ^b^
	36	0.14 ± 0.07 ^b^	0.61 ± 0.12 ^a^	0.17 ± 0.16 ^b^
GSH-PX	6	8.65 ± 2.99 ^a^	16.97 ± 3.73 ^a^	13.75 ± 5.59 ^a^
	24	14.34 ± 6.64 ^a^	10.43 ± 4.99 ^a^	12.62 ± 4.01 ^a^
	36	15.94 ± 1.83 ^b^	10.23 ± 2.23 ^c^	28.49 ± 2.28 ^a^

Notes: malondialdehyde (MDA); superoxide dismutase (SOD); catalase (CAT); glutathione peroxidase (GSH-PX). The letters annotated by peers indicate significant differences between different ammonia concentrations at the same time (*p* < 0.05).

## Data Availability

The original contributions presented in this study are included in the article. Further inquiries can be directed to the corresponding authors.

## Supplementary Materials

The following supporting information can be downloaded at: https://www.mdpi.com/article/10.3390/antiox13111357/s1, Table S1: Levene’s test for equality of error variances on malondialdehyde (MDA) concentration in the gills by time and ammonia nitrogen concentration; Table S2: Two-Way ANOVA of time and ammonia nitrogen concentration on malondialdehyde (MDA) concentration in the gills; Table S3. Levene’s test for equality of error variances on superoxide dismutase (SOD) activity in the gills by time and ammonia nitrogen concentration; Table S4. Two-Way ANOVA of time and ammonia nitrogen concentration on superoxide dismutase (SOD) activity in the gills; Table S5. Levene’s test for equality of error variances on catalase (CAT) activity in the gills by time and ammonia nitrogen concentration; Table S6. Two-Way ANOVA of time and ammonia nitrogen concentration on catalase (CAT) activity in the gills; Table S7. Levene’s test for equality of error variances on glutathione peroxidase (GSH-PX) activity in the gill by time and ammonia nitrogen concentration; Table S8. Two-Way ANOVA of time and ammonia nitrogen concentration on glutathione peroxidase (GHS-PX) activity in the gills; Table S9. Levene’s test for equality of error variances on malondialdehyde (MDA) concentration in the red muscle by time and ammonia nitrogen concentration; Table S10. Two-Way ANOVA of time and ammonia nitrogen concentration on malondialdehyde (MDA) concentration in the red muscle; Table S11. Levene’s test for equality of error variances on superoxide dismutase (SOD) activity in the red muscle by time and ammonia nitrogen concentration; Table S12. Two-Way ANOVA of time and ammonia nitrogen concentration on superoxide dismutase (SOD) activity in the red muscle; Table S13. Levene’s test for equality of error variances on catalase (CAT) activity in the red muscle by time and ammonia nitrogen concentration; Table S14. Two-Way ANOVA of time and ammonia nitrogen concentration on catalase (CAT) activity in the red muscle; Table S15. Levene’s test for equality of error variances on glutathione peroxidase (GHS-PX) activity in the red muscle by time and ammonia nitrogen concentration; Table S16. Two-Way ANOVA of time and ammonia nitrogen concentration on glutathione peroxidase (GHS-PX) activity in the red muscle; Table S17. Levene’s test for equality of error variances on malondialdehyde (MDA) concentration in the white muscle by time and ammonia nitrogen concentration; Table S18. Two-Way ANOVA of time and ammonia nitrogen concentration on malondialdehyde (MDA) concentration in the white muscle; Table S19. Levene’s test for equality of error variances on superoxide dismutase (SOD) activity in the white muscle by time and ammonia nitrogen concentration; Table S20. Two-Way ANOVA of time and ammonia nitrogen concentration on superoxide dismutase (SOD) activity in the white muscle; Table S21. Levene’s test for equality of error variances on catalase (CAT) activity in the white muscle by time and ammonia nitrogen concentration; Table S22. Two-Way ANOVA of time and ammonia nitrogen concentration on catalase (CAT) activity in the white muscle; Table S23. Levene’s test for equality of error variances on glutathione peroxidase (GHS-PX) activity in the white muscle by time and ammonia nitrogen concentration; Table S24. Two-Way ANOVA of time and ammonia nitrogen concentration on glutathione peroxidase (GHS-PX) activity in the white muscle.
