# Supplementary material for: The Impact of Acute Ammonia Nitrogen Stress on the Gill Tissue Structure and Antioxidant Ability of Gills and Red and White Muscle in Juvenile Yellowfin Tuna (Thunnus albacares)
Source: Antioxidants (Basel). 2024 Nov 6;13(11):1357. doi: 10.3390/antiox13111357 (PMC11591170; doi:10.3390/antiox13111357)
Supplement: Supplementary file 1 [file antioxidants-13-01357-s001.zip › antioxidants-3200004-supplementary.pdf]

Table S1. Levene's test for equality of error variances on malondialdehyde (MDA) concentration in the gills by time and ammonia nitrogen concentration.

Table S1 Levene's Test for Equality of Error Variances [a, b]

		Levene's Statistic	Df 1	Df 2	P
gill MDA concentration	Based on Mean	2.412	8	18	0.058
	Based on Median	2.160	8	18	0.083
	Based on Median with Adjusted Degrees of Freedom	2.160	8	6.104	0.180
	Based on Trimmed Mean	2.408	8	18	0.058

Test the null hypothesis of "equal error variances of the dependent variable across groups."

a. Dependent Variable: Gill MDA concentration

b. Model: Intercept + Time + Concentration + Time * Concentration

Table S2. Two-Way ANOVA of time and ammonia nitrogen concentration on malondialdehyde (MDA) concentration in the gills.

Table S2 Two-way ANOVA

Dependent variable: gill MDA concentration

Source	SS	Df	MS	F	P
Adjusted model	344.425[a]	8	43.053	2303.013	0.000
Intercept	1950.667	1	1950.667	104345.639	0.000
Stress time	220.900	2	110.450	5908.224	0.000
Ammonia nitrogen concentration	12.467	2	6.234	333.455	0.000
Stress time * Ammonia nitrogen concentration	111.058	4	27.765	1485.187	0.000
Error	0.336	18	0.019		
Total	2295.429	27			
Adjusted statistics	344.762	26			

a. $R^2 = 0.999$（Adjusted $R^2 = 0.999$）

Table S3. Levene's test for equality of error variances on superoxide dismutase (SOD) activity in the gills by time and ammonia nitrogen concentration.

Table S3 Levene's Test for Equality of Error Variances [a, b]

		Levene's Statistic	Df 1	Df 2	P
gill SOD activity	Based on Mean	1.284	8	18	0.311
	Based on Median	0.390	8	18	0.912
	Based on	0.390	8	11.124	0.904

Median with Adjusted Degrees of Freedom				
Based on Trimmed Mean	1.198	8	18	0.354

Test the null hypothesis of "equal error variances of the dependent variable across groups."

a. Dependent Variable: Gill SOD activity

b. Model: Intercept + Time + Concentration + Time * Concentration

Table S4. Two-Way ANOVA of time and ammonia nitrogen concentration on superoxide dismutase (SOD) activity in the gills.

Table S4 Two-way ANOVA

Dependent variable: gill SOD activity

Source	SS	Df	MS	F	P
Adjusted model	175.638	8	21.955	8.089	0.000
Intercept	1999.477	1	1999.477	736.645	0.000
Stress time	19.299	2	9.649	3.555	0.050
Ammonia nitrogen concentration	41.227	2	20.614	7.594	0.004
Stress time * Ammonia nitrogen concentration		4	28.778	10.602	0.000
Error		18	2.714		
Total		27			
Adjusted statistics		26			

a. $R^2 = 0.782$（Adjusted $R^2 = 0.686$）

Table S5. Levene's test for equality of error variances on catalase (CAT) activity in the gills by time and ammonia nitrogen concentration.

Table S5 Levene's Test for Equality of Error Variances [a, b]

		Levene's Statistic	Df 1	Df 2	P
gill CAT activity	Based on Mean	2.231	8	18	0.075
	Based on Median	0.344	8	18	0.937
	Based on Median with Adjusted Degrees of Freedom	0.344	8	6.180	0.918
	Based on Trimmed Mean	1.980	8	18	0.109

Test the null hypothesis of "equal error variances of the dependent variable across groups."

a. Dependent Variable: Gill CAT activity

b. Model: Intercept + Time + Concentration + Time * Concentration

Table S6. Two-Way ANOVA of time and ammonia nitrogen concentration on catalase (CAT) activity in the gills.

Table S6 Two-way ANOVA

Dependent variable: gill CAT activity

Source	SS	Df	MS	F	P
Adjusted model	1811.953[a]	8	226.494	99.255	0.000
Intercept	3922.238	1	3922.238	1718.808	0.000
Stress time	833.250	2	416.625	182.574	0.000
Ammonia nitrogen concentration	372.002	2	186.001	81.509	0.000
Stress time * Ammonia nitrogen concentration	606.701	4	151.675	66.467	0.000
Error	41.075	18	2.282		
Total	5775.266	27			
Adjusted statistics	1853.028	26			

a. $R^2 = 0.978$ （Adjusted $R^2 = 0.968$）

Table S7. Levene's test for equality of error variances on glutathione peroxidase (GSH-PX) activity in the gill by time and ammonia nitrogen concentration.

Table S7 Levene's Test for Equality of Error Variances [a, b]

		Levene's Statistic	Df 1	Df 2	P
gill GSH-PX activity	Based on Mean	2.516	8	18	0.050
	Based on Median	0.874	8	18	0.555
	Based on Median with Adjusted Degrees of Freedom	0.874	8	9.629	0.568
	Based on Trimmed Mean	2.368	8	18	0.061

Test the null hypothesis of "equal error variances of the dependent variable across groups."

a. Dependent Variable: Gill GSH-PX activity

b. Model: Intercept + Time + Concentration + Time * Concentration

Table S8. Two-Way ANOVA of time and ammonia nitrogen concentration on glutathione peroxidase (GHS-PX) activity in the gills.

Table S8 Two-way ANOVA

Dependent variable: gill GSH-PX activity

Source	SS	Df	MS	F	P
Adjusted model	16104.876[a]	8	2013.109	35.833	0.000

Intercept	184969.397	1	184969.397	3292.417	0.000
Stress time	4397.218	2	2198.609	39.135	0.000
Ammonia nitrogen concentration	8235.497	2	4117.749	73.295	0.000
Stress time * Ammonia nitrogen concentration	3472.161	4	868.040	15.451	0.000
Error	1011.248	18	56.180		
Total	202085.521	27			
Adjusted statistics	17116.123	26			

a. R^2 = .941（Adjusted R^2 = 0.915）

Table S9. Levene's test for equality of error variances on malondialdehyde (MDA) concentration in the red muscle by time and ammonia nitrogen concentration.

Table S9 Levene's Test for Equality of Error Variances [a, b]

		Levene's Statistic	Df 1	Df 2	P
red muscle MDA concentration	Based on Mean	1.984	8	18	0.108
	Based on Median	0.907	8	18	0.532
	Based on Median with Adjusted Degrees of Freedom	0.907	8	8.050	0.553
	Based on Trimmed Mean	1.909	8	18	0.121

Test the null hypothesis of "equal error variances of the dependent variable across groups."

a. Dependent Variable: Red muscle MDA concentration

b. Model: Intercept + Time + Concentration + Time * Concentration

Table S10. Two-Way ANOVA of time and ammonia nitrogen concentration on malondialdehyde (MDA) concentration in the red muscle.

Table S10 Two-way ANOVA

Dependent variable: red muscle MDA concentration

Source	SS	Df	MS	F	P
Adjusted model	2.232[a]	8	0.279	162.551	0.000
Intercept	18.784	1	18.784	10944.778	0.000
Stress time	0.423	2	0.211	123.198	0.000
Ammonia nitrogen concentration	0.005	2	0.003	1.579	0.233
Stress time * Ammonia nitrogen concentration	1.804	4	0.451	262.714	0.000
Error	0.031	18	0.002		

Total	21.046	27
Adjusted statistics	2.263	26

a. R^2 = .986（Adjusted R^2 = 0.980）

Table S11. Levene's test for equality of error variances on superoxide dismutase (SOD) activity in the red muscle by time and ammonia nitrogen concentration.

Table S11 Levene's Test for Equality of Error Variances [a, b]

		Levene's Statistic	Df 1	Df 2	P
red muscle SOD activity	Based on Mean	1.203	8	18	0.351
	Based on Median	0.802	8	18	0.609
	Based on Median with Adjusted Degrees of Freedom	0.802	8	11.578	0.613
	Based on Trimmed Mean	1.180	8	18	0.363

Test the null hypothesis of "equal error variances of the dependent variable across groups."

a. Dependent Variable: Red muscle SOD activity

b. Model: Intercept + Time + Concentration + Time * Concentration

Table S12. Two-Way ANOVA of time and ammonia nitrogen concentration on superoxide dismutase (SOD) activity in the red muscle.

Table S12 Two-way ANOVA

Dependent variable: red muscle SOD activity

Source	SS	Df	MS	F	P
Adjusted model	2.212[a]	8	0.276	22.227	0.000
Intercept	7.614	1	7.614	612.160	0.000
Stress time	1.516	2	0.758	60.946	0.000
Ammonia nitrogen concentration	0.391	2	0.196	15.730	0.000
Stress time * Ammonia nitrogen concentration	0.304	4	0.076	6.117	0.003
Error	0.224	18	0.012		
Total	10.050	27			
Adjusted statistics	2.436	26			

a. R^2 = 0.908（Adjusted R^2 = 0.867）

Table S13. Levene's test for equality of error variances on catalase (CAT) activity in the red muscle by time and ammonia nitrogen concentration.

Table S13 Levene's Test for Equality of Error Variances [a, b]

		Levene's Statistic	Df 1	Df 2	P

red muscle CAT activity	Based on Mean	2.346	8	18	0.063
	Based on Median	0.582	8	18	0.780
	Based on Median with Adjusted Degrees of Freedom	0.582	8	8.913	0.771
	Based on Trimmed Mean	2.155	8	18	0.084

Test the null hypothesis of "equal error variances of the dependent variable across groups."

a. Dependent Variable: Red muscle CAT activity

b. Model: Intercept + Time + Concentration + Time * Concentration

Table S14. Two-Way ANOVA of time and ammonia nitrogen concentration on catalase (CAT) activity in the red muscle.

Table S14 Two-way ANOVA

Dependent variable: red muscle CAT activity

Source	SS	Df	MS	F	P
Adjusted model	3.547[a]	8	0.443	29.519	0.000
Intercept	21.286	1	21.286	1417.267	0.000
Stress time	0.831	2	0.416	27.677	0.000
Ammonia nitrogen concentration	1.188	2	0.594	39.543	0.000
Stress time * Ammonia nitrogen concentration	1.528	4	0.382	25.428	0.000
Error	0.270	18	0.015		
Total	25.103	27			
Adjusted statistics	3.817	26			

a. R^2 = 0.929（Adjusted R^2 = 0.898）

Table S15. Levene's test for equality of error variances on glutathione peroxidase (GHS-PX) activity in the red muscle by time and ammonia nitrogen concentration.

Table S15 Levene's Test for Equality of Error Variances [a, b]

		Levene's Statistic	Df 1	Df 2	P
red muscle GSH-PX activity	Based on Mean	2.094	8	18	0.092
	Based on Median	0.755	8	18	0.645
	Based on Median with Adjusted Degrees of Freedom	0.755	8	11.218	0.647
	Based on	1.979	8	18	0.109

	Trimmed Mean				

Test the null hypothesis of "equal error variances of the dependent variable across groups."

a. Dependent Variable: Red muscle GSH-PX activity

b. Model: Intercept + Time + Concentration + Time * Concentration

Table S16. Two-Way ANOVA of time and ammonia nitrogen concentration on glutathione peroxidase (GHS-PX) activity in the red muscle.

Table S16 Two-way ANOVA

Dependent variable: red muscle GSH-PX activity

Source	SS	Df	MS	F	P
Adjusted model	208.849[a]	8	26.106	74.200	0.000
Intercept	1158.665	1	1158.665	3293.202	0.000
Stress time	24.107	2	12.054	34.259	0.000
Ammonia nitrogen concentration	54.640	2	27.320	77.649	0.000
Stress time * Ammonia nitrogen concentration	130.102	4	32.525	92.445	0.000
Error	6.333	18	0.352		
Total	1373.847	27			
Adjusted statistics	215.182	26			

a. R^2 = 0.971（Adjusted R^2 = 0.957）

Table S17. Levene's test for equality of error variances on malondialdehyde (MDA) concentration in the white muscle by time and ammonia nitrogen concentration.

Table S17 Levene's Test for Equality of Error Variances [a, b]

		Levene's Statistic	Df 1	Df 2	P
white muscle MDA concentration	Based on Mean	2.256	8	18	0.072
	Based on Median	0.385	8	18	0.915
	Based on Median with Adjusted Degrees of Freedom	0.385	8	7.723	0.900
	Based on Trimmed Mean	2.022	8	18	0.102

Test the null hypothesis of "equal error variances of the dependent variable across groups."

a. Dependent Variable: White muscle MDA concentration

b. Model: Intercept + Time + Concentration + Time * Concentration

Table S18. Two-Way ANOVA of time and ammonia nitrogen concentration on malondialdehyde (MDA) concentration in the white muscle.

Table S18 Two-way ANOVA

Dependent variable: white muscle MDA concentration

Source	SS	Df	MS	F	P
Adjusted model	0.131[a]	8	0.016	21.363	0.000
Intercept	3.958	1	3.958	5167.687	0.000
Stress time	0.038	2	0.019	25.110	0.000
Ammonia nitrogen concentration	0.052	2	0.026	33.833	0.000
Stress time * Ammonia nitrogen concentration	0.041	4	0.010	13.254	0.000
Error	0.014	18	0.001		
Total	4.102	27			
Adjusted statistics	0.145	26			

a. R^2 = 0. 905（Adjusted R^2 = 0. 862）

Table S19. Levene's test for equality of error variances on superoxide dismutase (SOD) activity in the white muscle by time and ammonia nitrogen concentration.

Table S19 Levene's Test for Equality of Error Variances [a, b]

		Levene's Statistic	Df 1	Df 2	P
white muscle SOD activity	Based on Mean	1.719	8	18	0.162
	Based on Median	0.303	8	18	0.955
	Based on Median with Adjusted Degrees of Freedom	0.303	8	7.511	0.943
	Based on Trimmed Mean	1.552	8	18	0.208

Test the null hypothesis of "equal error variances of the dependent variable across groups."

a. Dependent Variable: white muscle SOD activity

b. Model: Intercept + Time + Concentration + Time * Concentration

Table S20. Two-Way ANOVA of time and ammonia nitrogen concentration on superoxide dismutase (SOD) activity in the white muscle.

Table S20 Two-way ANOVA

Dependent variable: white muscle SOD activity

Source	SS	Df	MS	F	P
Adjusted model	0.030[a]	8	0.004	3.422	0.014
Intercept	1.410	1	1.410	1281.837	0.000
Stress time	0.012	2	0.006	5.411	0.014
Ammonia nitrogen concentration	0.005	2	0.003	2.363	0.123

Stress time * Ammonia nitrogen concentration	0.013	4	0.003	2.958	0.048
Error	0.020	18	0.001		
Total	1.460	27			
Adjusted statistics	0.050	26			

a. $R^2 = 0.603$（Adjusted $R^2 = 0.427$）

Table S21. Levene's test for equality of error variances on catalase (CAT) activity in the white muscle by time and ammonia nitrogen concentration.

Table S21 Levene's Test for Equality of Error Variances [a, b]

		Levene's Statistic	Df 1	Df 2	P
white muscle CAT activity	Based on Mean	2.164	8	18	0.083
	Based on Median	0.673	8	18	0.709
	Based on Median with Adjusted Degrees of Freedom	0.673	8	9.455	0.706
	Based on Trimmed Mean	2.018	8	18	0.103

Test the null hypothesis of "equal error variances of the dependent variable across groups."

a. Dependent Variable: White muscle CAT activity

b. Model: Intercept + Time + Concentration + Time * Concentration

Table S22. Two-Way ANOVA of time and ammonia nitrogen concentration on catalase (CAT) activity in the white muscle.

Table S22 Two-way ANOVA

Dependent variable: white muscle CAT activity

Source	SS	Df	MS	F	P
Adjusted model	2.272[a]	8	0.284	9.335	0.000
Intercept	4.202	1	4.202	138.118	0.000
Stress time	0.560	2	0.280	9.209	0.002
Ammonia nitrogen concentration	0.701	2	0.350	11.517	0.001
Stress time * Ammonia nitrogen concentration	1.011	4	0.253	8.306	0.001
Error	0.548	18	0.030		
Total	7.021	27			
Adjusted statistics	2.819	26			

a. $R^2 = 0.806$（Adjusted $R^2 = 0.719$）

Table S23. Levene's test for equality of error variances on glutathione peroxidase (GHS-PX) activity in the white muscle by time and ammonia nitrogen concentration.

Table S23 Levene's Test for Equality of Error Variances [a, b]

		Levene's Statistic	Df 1	Df 2	P
white muscle GSH-PX activity	Based on Mean	1.674	8	18	0.173
	Based on Median	0.339	8	18	0.939
	Based on Median with Adjusted Degrees of Freedom	0.339	8	11.465	0.933
	Based on Trimmed Mean	1.510	8	18	0.222

Test the null hypothesis of "equal error variances of the dependent variable across groups."

a. Dependent Variable: White muscle GSH-PX activity

b. Model: Intercept + Time + Concentration + Time * Concentration

Table S24. Two-Way ANOVA of time and ammonia nitrogen concentration on glutathione peroxidase (GHS-PX) activity in the white muscle.

Table S24 Two-way ANOVA

Dependent variable: white muscle GSH-PX activity

Source	SS	Df	MS	F	P
Adjusted model	831.376[a]	8	103.922	6.125	0.001
Intercept	5758.161	1	5758.161	339.387	0.000
Stress time	178.829	2	89.415	5.270	0.016
Ammonia nitrogen concentration	184.066	2	92.033	5.424	0.014
Stress time * Ammonia nitrogen concentration	468.481	4	117.120	6.903	0.001
Error	305.394	18	16.966		
Total	6894.931	27			
Adjusted statistics	1136.770	26			

a. R^2 = 0.731（Adjusted R^2 = 0.612）
